# Hospital in Home COVID-19 Monitoring: A Novel Approach to Keeping a Watchful Eye on Older Adults

**DOI:** 10.1093/geroni/igab046.2705

**Published:** 2021-12-17

**Authors:** Alissa Cooney, Monica Serra, Monica Lee, Kimberly Oakman, Laura Medlin, Sunil Ahuja, Marcos Restrepo, Sandra Sanchez-Reilly

**Affiliations:** 1 University of Texas HSC San Antonio, San Antonio, Texas, United States; 2 UT Health San Antonio - Barshop Institute, San Antonio, Texas, United States; 3 South Texas Veterans Health Care System, San Antonio, Texas, United States

## Abstract

Older adults suffering from severe acute respiratory syndrome coronavirus 2 (SARS-CoV-2) are at increased risk of death and hospitalization-related complications. The coronavirus disease 2019 (COVID-19) pandemic has forced adaptations in Telehealth, allowing COVID-19 patients to be managed at home. Traditionally, Hospital in Home (HIH) patients have better clinical outcomes and lower mortality compared to similar hospitalized patients. However, effectiveness of HIH for COVID-19 older adults remains unknown. This study examines the effect of age on rates of hospital readmission and overall mortality for patients enrolled in HIH after initial COVID-19 hospital discharge. A HIH COVID-19 monitoring program was developed to facilitate earlier hospital discharge and monitoring. Retrospective data between March 2020 and January 2021 were analyzed. Of the 402 subjects (age:26-99; mean:61.8), 13 (6.1%) subjects <65 years old vs 19 (10%) subjects □65 years old were readmitted to the hospital at least once. Two (0.94%) subjects <65 years old and 12 (6.3%) subjects □65 years old died. Older adults were 1.719 times more likely to be re-hospitalized (p=0.005) and 7.153 times more likely to die (p=0.017) compared to younger adults. Age remains a significant predictor of hospital readmission and mortality in subjects previously hospitalized for COVID-19 even when followed by monitoring programs like HIH. Further studies are needed to determine the best way to reduce hospital readmission and mortality rates for older adults after initial COVID-19 hospital discharge.

